# Emergency Surgery for Metastatic Melanoma

**DOI:** 10.1155/2014/987170

**Published:** 2014-10-28

**Authors:** Dimitrios Mantas, Petros Tsaparas, Petros Charalampoudis, Helen Gogas, Gregory Kouraklis

**Affiliations:** ^1^2nd Propaedeutic Department of Surgery, Laiko General Hospital, Athens University School of Medicine, 17 Agiou Thoma Street, 11527 Athens, Greece; ^2^1st Internal Medicine Department, Laiko General Hospital, Athens University School of Medicine, 17 Agiou Thoma Street, 11527 Athens, Greece

## Abstract

Visceral metastases from malignant melanoma (stage M1c) confer a very poor prognosis, as documented on the most recent revised version of the TNM/AJCC staging system. Emergency surgery for intra-abdominal complications from the disease is rare. We report on our 5-year single institution experience with surgical management of metastatic melanoma to the viscera in the emergent setting. From 2009 to 2013, 14 patients with metastatic melanoma were admitted emergently due to an acute abdomen. Clinical manifestations encompassed intestinal obstruction and bleeding. Surgical procedures involved multiple enterectomies with primary anastomoses in 8 patients, and one patient underwent splenectomy, one adrenalectomy, one right colectomy, one gastric wedge resection, one gastrojejunal anastomosis, and one transanal debulking, respectively. The 30-day mortality was 7 percent. Median follow-up was 14 months. Median overall survival was 14 months. Median disease free survival was 7.5 months. One-year overall survival was 64.2 percent and 2-year overall survival was 14.2 percent. Emergency surgery for metastatic melanoma to the viscera is rare. Elective curative surgery combined with novel cytotoxic systemic therapies is under investigation in an attempt to grant survival benefit in melanoma patients with visceral disease.

## 1. Introduction

Metastatic melanoma is an aggressive disease with dismal prognosis despite novel chemotherapeutic agents. M1c disease is defined as the presence of visceral metastases with elevated lactate dehydrogenase (LDH) level in the absence of pulmonary metastasis and carries a median survival of about 6 to 10 months [1]. Advanced melanoma can disseminate to any organ, with the commonest distant sites of metastasis being the skin, lung, and brain. In cases of visceral involvement, metastatic malignant melanoma most commonly attains the liver and the small bowel. Interestingly, up to 95 percent of patients with metastases to the gastrointestinal tract will not be identified until autopsy [1]. Intra-abdominal dissemination of melanoma can manifest itself with weight loss, vague abdominal pain, and/or anemia. In some cases, patients can present in the emergency setting with bowel intussusception, obstruction, bleeding, or perforation [2]. Although an acute abdominal symptomatology can raise the suspicion of intra-abdominal metastasis in any patient with a history of cutaneous melanoma, diagnosis is definitely made at surgery. We report on our five-year, single-center experience in emergency surgery for M1c melanoma.

## 2. Case Series

Between 2009 and 2013, 14 consecutive patients (9 men and 5 women) with M1c melanoma underwent emergency surgical exploration due to acute abdomen by a single senior consultant general surgeon (DM). Clinical characteristics were as follows: all patients were Caucasian with a history of known metastatic melanoma and had previous chemotherapy for palliation. Median age was 61.5 years old (range of 17–87). An elevated serum lactate dehydrogenase (LDH) level was documented in 8 out of 14 patients (LDH mean value 652.6 U/L, range of 251–1567, Table 1). A senior consultant oncologist would be following all patients at the time of presentation to the acute setting.

## 3. Surgery

All patients were admitted emergently in the Surgical Department. Acute abdominal pain was reported as the chief complaint. All patients underwent diagnostic abdominal computed tomography preoperatively in order to confirm the clinical suspicion as well as to acquire information on the disease extent and intra-abdominal localization. Intraoperative data were as follows: all patients were operated approximately 3 to 4 hours after admission. Intestinal obstruction was the commonest intraoperative finding (9 cases) followed by intra-abdominal bleeding (5 cases). The type of surgery varied significantly; 8 out of 14 patients underwent multiple enterectomies with primary end-to-end enteroenteral anastomoses (range of 2–5 resections); one patient underwent a right hemicolectomy with primary ileotransverse anastomosis due to ileocecal valve involvement by the disease; one patient underwent splenectomy for splenic rupture due to splenic metastasis; one patient underwent wedge resection of a gastric lesion; one patient underwent a gastrojejunal bypass procedure for gastrointestinal bleeding; one patient underwent laparoscopic exploration with left adrenalectomy due to adrenal rupture; one patient who presented with bleeding per rectum and rectal perforation underwent a transanal rectal tumor debulking; in this latter case, we opted for limited palliative surgery over formal abdominoperineal resection due to high intraoperative morbidity.

## 4. Outcomes

Following surgery, two patients developed early intestinal obstruction and underwent reoperation and one patient was admitted with late obstruction in another tertiary center. One of them died in the immediate postoperative period; the 30-day mortality was 7.14 percent. We documented a 14.28 percent cumulative operative morbidity in the form of intestinal obstruction. All histopathology reports were consistent with metastatic melanoma. Median follow-up was 14 months (range of 0.5–26). Recurrence events pertained mainly to peritoneum, brain, and lung. Median disease free survival was 7.5 months (range of 0–26). One-year overall survival was 64.2 percent and 2-year overall survival was 14.2 percent.

## 5. Discussion

Metastatic melanoma carries a very dismal prognosis. The American Joint Committee on Cancer (AJCC, 2007 Revision) has divided patients with systemic disease into three groups: M1a (distant skin, subcutaneous, or nodal metastases), M1b (lung metastases), and M1c (absence of pulmonary lesion with any visceral or distant metastases and an elevated LDH level). Cumulative evidence suggests that M1a disease has the best survival rates, while M1c has the worst [1, 3]. In Europe melanoma shows some significant geographical heterogeneity, with the highest rates being reported in Scandinavia (15 cases/100,000 per year) and the lowest in the Mediterranean basin (7 cases/100,000 per year) [4]. About 20 percent of patients with melanoma will ultimately develop distant metastases; prognosis remains poor despite novel treatment modalities; single-agent or multiple-agent chemotherapy, biological therapy (interferon-alpha, interleukin-2), radiotherapy, and biochemotherapy have failed to demonstrate a consistent survival benefit in advanced systemic disease [5].

Surgery for stage IV melanoma has demonstrated debatable results. In carefully selected patients, elective surgery can improve quality of life albeit it grants no survival benefit [6–11]. Conversely, Wasif and coworkers compared surgery with no surgery for metastatic melanoma on 4229 patients. They concluded that patients who underwent metastasectomy (33.6 percent) showed improved median and 5-year overall survival rates compared to patients who were ineligible for resection [12]; notably, metastasectomy was found to be an independent predictor of survival in this study.

Emergency surgery on the small bowel can be employed for a variety of reasons. Most commonly, intestinal obstruction, perforation, and intractable bleeding can lead urgently a patient to the operating theatre. Adhesions, tumors, diverticulosis, neoplasia, inflammatory bowel disease, and acute ischemia account for the majority of emergency surgical admissions pertaining to the small bowel [13–17]. Cutaneous melanoma exhibits an unusual predilection for metastasizing to the small intestine causing vague abdominal pain and weight loss. At autopsy, metastatic deposits are reportedly present in 50 to 60 percent of patients with melanoma; however less than 4 percent of patients are found to have gastrointestinal metastasis during the course of their disease; moreover, in up to 9 percent of cases of gastrointestinal melanoma the primary site is unidentifiable. Nevertheless, gastrointestinal melanoma rarely manifests itself with acute abdominal symptoms and emergency surgery has scarcely been reported [16, 18, 19]. Bleeding, obstruction, perforation, and intussusception are the leading causes that prompt emergency surgical management. Generally, intestinal metastasis of melanoma is difficult to diagnose and when encountered it reflects advanced disease and dismal outcome [16, 19, 20].

Spontaneous splenic rupture is a rare cause of major nontraumatic hemorrhage and usually occurs in cases that are infectious insults to the spleen [21]. Acute abdomen during the course of treatment for melanoma has been rarely attributed to pharmacologic agents [22, 23]. Next to the spleen, the adrenal can also represent a site of solitary metastasis from melanoma [24]. Complete adrenal resection and adjuvant treatment have been advocated in such cases. Rare reports of metastatic melanoma to the stomach, colon, and rectum/anal canal have been also reported [2, 25, 26].

A rigorous approach by a multidisciplinary team is crucial in an attempt to deliver the best of care in cases of metastatic melanoma. Metastatic melanoma to the gastrointestinal tract or to another solid organ should be suspected in any patient with a history of cutaneous melanoma and a new onset of abdominal symptoms. Recently, new biological therapies such as vemurafenib and ipilimumab have shown promising results in IV melanoma [27].

## 6. Conclusion

Surgery with a curative intent for stage IV melanoma remains debatable. A combination of modern systemic therapies and surgical resection is currently under investigation. A better understanding of tumor biology is essential in designing new strategies. Surgery for M1c disease could possibly gain ground in the future by debulking a significant load of disease and therefore by giving way to more aggressive and efficient systemic treatments.

## Figures and Tables

**Table 1:** Clinicopathological characteristics, operative data, and outcomes.

Number	Gender	Age	LDH	Adj Tx	Clinical presentation	GI site	Primary	Extra GI metastasis	Surgery	Postoperative course	Follow-up (months)	DFS	Recurrence	OS
1	F	71	Increased	Yes	Obstruction	Jejunum, ileum	Hyponychium	No	3 enterectomies	Uneventful	16	9	Peritoneum, brain	16
2	M	85	Normal	Yes	Obstruction	Ileum	Back	No	Enterectomy	Uneventful	18	12	Peritoneum, lung	18
3	M	33	Increased	Yes	Obstruction	Jejunum, ileum	Lower extremity	No	2 enterectomies	Uneventful	10	6	Brain	10
4	F	66	Increased	Yes	Obstruction	Ileum	Back	No	2 enterectomies	Uneventful	19	11	Lung	19
5	M	61	Increased	Yes	Obstruction	Ileum	Thigh	Bladder	3 enterectomies	Uneventful	9	0	Peritoneum	9
6	F	57	Increased	Yes	Obstruction	Ileum	Sole	Omentum	Enterectomy	Uneventful	11	0	Peritoneum	11
7	M	65	Normal	Yes	Obstruction	Ileum	Cutaneous	Peritoneum	Jejunum-transverse bypass	Obstruction reoperation	7	0	Peritoneum, brain	7
8	M	66	Increased	Yes	Obstruction	Ileum	Upper extremity	No	Enterectomy	Uneventful	24	20	Brain	24
9	F	52	Increased	Yes	Obstruction	Ileum	Cutaneous	No	Multiple enterectomies	Obstruction reoperation	1	0	Peritoneum	0,5
10	M	17	Increased	Yes	Obstruction	Ileocecal valve	Back	No	Right hemicolectomy	Uneventful	14	6	Brain	14
11	F	47	Increased	Yes	Bleeding	Adrenal	Lower extremity	No	Lap. adrenalectomy	Obstruction reoperation	26	26	No	26
12	M	68	Increased	Yes	Bleeding	Spleen	Back	No	Splenectomy	Uneventful	14	12	Peritoneum	14
13	M	65	Increased	Yes	Bleeding	Stomach	Back	No	Wedge resection	Uneventful	22	19	Peritoneum	22
14	M	50	Increased	Yes	Bleeding	Rectum	Upper extremity	No	Transanal resection	Uneventful	3	0	Local, brain	3

Total: 14 patients, median age: 63 years (17–85), median follow-up: 14 months (0–26), median DFS: 7 (0.5–26), median OS: 14 months, 1 y OS: 64.20%, and 2 y OS: 14.20%.
